# Level and timing of product substitution in a trial of e-cigarettes for smokers not interested in quitting

**DOI:** 10.18332/tid/189220

**Published:** 2024-06-13

**Authors:** James D. Sargent, Sarah I. Pratt, Mary F. Brunette, Joelle C. Ferron, Meghan M. Santos, Mike Stoolmiller

**Affiliations:** 1 Department of Pediatrics, Geisel School of Medicine, Dartmouth College, Hanover, United States; 2 Department of Biomedical Data Sciences, Geisel School of Medicine, Dartmouth College, Hanover, United States; 3 Department of Psychiatry, Dartmouth Hitchcock Medical Center, Lebanon, United States; 4 Boston University Wheelock College of Education and Human Development, Boston, United States

**Keywords:** harm reduction, electronic cigarettes, methods, switching trial

## Abstract

**INTRODUCTION:**

The e-cigarette market is large and diverse. Traditional smoking cessation trials involving a control group and a 6-month observation period are an inefficient methodology for testing the multiple treatment options e-cigarettes provide for harm reduction in cigarette smokers. We determined when product substitution occurred in the e-cigarette provision arm of an e-cigarette substitution trial for cigarette smokers who were not interested in quitting.

**METHODS:**

We conducted a secondary analysis of 120 cigarette smokers with severe mental illness (recruitment 2017–2020) who were given disposable e-cigarettes for 8 weeks and assessed at weeks 0 (t0), 2, 4, 6, and 8. We explored product substitution through visit-to-visit correlations in change in product use, then developed a dual process growth model for cigarette and e-cigarette use to test the association between increases in e-cigarette use and concurrent decreases in cigarettes smoked.

**RESULTS:**

Mean age of the participants was 45.9 years, and 42.7% smoked ≥20 cigarettes per day. Almost all product substitution occurred between t0 and t2. For the average smoker (18 cigarettes per day), t2 cigarette frequency decreased by 0.39 (95% CI: -0.56 – -0.22) cigarettes for each additional e-cigarette session. There was effect modification (p=0.033), such that baseline light smokers (<10 cigarettes/day) had no significant decrease in t2 cigarette frequency, regardless of their initial increase in e-cigarette use, while heavy smokers (38 cigarettes/day) switched products nearly on a one-to-one basis.

**CONCLUSIONS:**

In this study, most product substitution occurred early, and heavier smokers had larger t2 decreases in cigarettes/day with increased e-cigarette use. If confirmed with replication studies, the findings could suggest establishment of a novel outcome for e-cigarette studies – early product substitution – and support the value of short-term comparative effectiveness trials that compare multiple potentially lower harm tobacco products.

**CLINICAL TRIAL REGISTRATION:**

The study was registered on the official website of ClinicalTrials.gov

**IDENTIFIER:**

ID NCT03050853

## INTRODUCTION

Limited toxicological evidence indicates that e-cigarettes may be a less harmful tobacco product that delivers nicotine with much lower levels of the cigarette smoke constituents that cause disease^1^. The potential short- and medium-term public health benefits of e-cigarettes depend on the degree to which they are: 1) taken up by established smokers – those at highest risk for smoking-related disease; and 2) substituted for most or all cigarettes^2^.

Several e-cigarette trials have been published in which cigarette smokers are asked to substitute these products for cigarettes. These trials have primarily focused on reductions in carbon monoxide and other biomarkers of harm at 4, 6, or 8 weeks^3-8^. Research has not closely examined patterns of product substitution – the relationship between e-cigarette uptake and cigarette reduction over time.

In a randomized trial with an assessment-only (control) condition, the e-cigarette as the cause of cigarette reductions can be inferred by comparing reductions in cigarettes across the group that received e-cigarettes and the group that did not. In the present study, we model the relationship between trajectories of e-cigarette and cigarette consumption within the e-cigarette arm of a randomized controlled trial that compared e-cigarette provision to assessment-only among smokers who were not already using e-cigarettes and had been unable to quit with standard cessation treatment^8^. As expected, the control group demonstrated very little change in cigarette consumption during the trial^8^. Given that cigarette smoking is a very consistent behavior within persons^9^, assessment-only groups in these sorts of trials may be unnecessary. Instead, comparative effectiveness trials could assign smokers to multiple different e-cigarette products to determine which assignment is most strongly associated with product substitution. The aim of this study is to explore the appropriate outcome and time course for such trials.

## METHODS

### Study overview

The data analyzed in this study come from a randomized controlled clinical trial (ClinicalTrials.gov ID NCT03050853) in which half of the enrolled adult smokers with severe mental illness were randomly assigned to be given e-cigarettes for 8 weeks and asked to substitute them for their cigarettes.

Their changes in cigarette use biomarkers (carbon monoxide and NNAL) were compared to an assessment-only control group. Persons with severe mental illness tend to be heavy smokers and have difficulty sustaining quit attempts and thus are an important user segment for harm reduction interventions^10^.

### Study sites

Participants were recruited from two urban mental health agencies (in Kentucky and Massachusetts) serving primarily Medicaid beneficiaries with severe mental illness. Trained research staff implemented the study protocol.

### Eligibility criteria

Participants were adults aged ≥18 years, enrolled in services for at least 3 months, and met criteria for DSM-V Axis I diagnosis of schizophrenia, schizoaffective disorder, or bipolar disorder and at least moderate impairment in multiple areas of psychosocial functioning. Eligibility also required that participants had smoked regularly for at least 5 years, were currently smoking at least 10 cigarettes per day with breath CO ≥10 ppm and had made at least one attempt to quit smoking in the past 5 years using evidence-based treatment, but were not currently interested in quitting. Individuals with psychiatric instability (hospitalization in the past month), active substance use disorder, current e-cigarette use (>4 times in the past month), or current pregnancy (or plans to become pregnant) were excluded.

### Intervention: E-cigarette provision

E-cigarettes were supplied for 8 weeks and data were collected for 26 weeks. The e-cigarette provision group had substantial reductions in mean cigarettes per day and CO during the 8-week period, but the reductions did not last after e-cigarette distribution ended. The main effects of the trial are reported elsewhere^8^.

This analysis involves only the e-cigarette arm of the trial. Study coordinators provided participants with a 2-week supply of e-cigarettes (matched to their baseline amount of smoking). Per product packaging, each disposable e-cigarette provided up to 300 puffs, roughly the equivalent of twenty cigarettes. Participants were given instructions on their safe use, choices of either tobacco or menthol flavored disposable e-cigarettes according to their typical cigarette use and started with 4.5% nicotine content, with the option to increase to 6.0% nicotine if needed. Participants were also given the opportunity to practice using the e-cigarette before leaving the appointment and they received additional 2-week supplies at 2, 4, and 6 weeks.

### Measures

An unblinded Study Coordinator used the Timeline Follow-Back method^11^, a structured interview that utilizes a calendar and participant-specific memory anchors to obtain self-reported cigarettes smoked and e-cigarette sessions each day over the past two weeks at 2, 4, 6, and 8 week assessments. This method has been shown to be reliable and valid to document substance use in the general population^12^ and among people with severe mental illness^12,13^. Participants were asked to bring used e-cigarettes back for a count at each visit, when breath CO was also obtained. Although latent constructs defined by using these additional variables might be more reliable, we elected to model only the self-report product frequency data for simplicity with a modest sample size and because of issues with the correlation structure for CO (many persons with severe mental illness also use cannabis^14^).

### Participant flow

Of the 959 individuals screened, 436 did not meet inclusion criteria, 133 could not be contacted, 106 declined to participate, and 44 consented but were not randomized (mostly screen failures), leaving 240 randomized participants for analysis. The characteristics of the 240 participants in the e-cigarette and control arms are described in the Supplementary file Table 1 (taken from the main effects article^8^). Of those, 210 (87.5%) were assessed at 8 weeks, of whom 107 were in the e-cigarette arm.

### Statistical analyses

Our goal was to study person-level substitution from cigarettes to e-cigarettes, which required examination of correlated change in the use of each product. Dual process growth models are a longstanding method for studying correlated change for two outcomes^15^. Dual process models with latent constructs are a type of random effects model that fits a mean growth trajectory for all observations in the data (the fixed effect), along with a unique growth curve for each individual in the data (the random effect)^14^. The original study was powered for an analysis of mean differences between the e-cigarette versus control arms across follow-up, not to detect correlated change in a dual process growth model, which requires a minimum of 4 correlated latent variables, 2 intercepts and 2 slopes for linear change.

**Figure 1:**
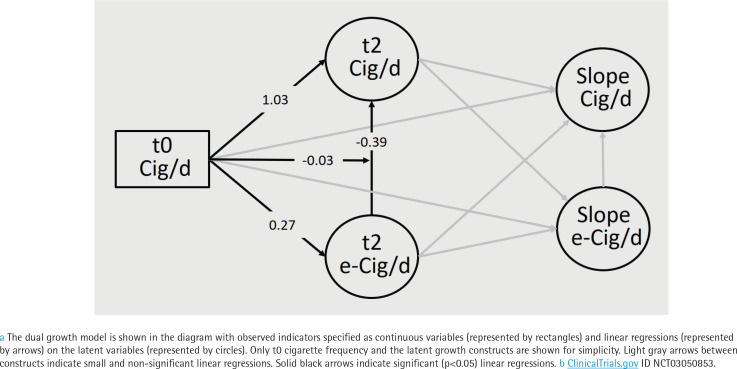
Dual growth model ^a^ for e-cigarettes and cigarettes, participants (N=120) in the treatment arm of the trial ^b^, recruitment period March 2017 through August 2020.

The dual growth model is shown in Figure 1, with observed indicators specified as continuous variables (represented by rectangles) and linear regressions (represented by arrows) on the latent variables (represented by circles). Only t0 cigarette frequency and the latent growth constructs are shown for simplicity. Light gray arrows between constructs indicate small and non-significant linear regressions, whereas solid black arrows indicate significant (p<0.05) linear regressions. The model was parameterized such that the intercept for each linear growth curve represented the t2 follow-up level. The encouragement of e-cigarette use dictated that within the dual process growth model, any cross-sectional relations would give causal priority to latent e-cigarette constructs predicting latent cigarette constructs. Thus, latent t2 e-cigarette frequency predicted latent t2 cigarette frequency and latent e-cigarette slope predicted latent cigarette slope. The rest of the structural relations respected temporal ordering.

The design element of enrolling only non-e-cigarette users in the trial meant that baseline e-cigarette use could be ignored due to lack of variance, and the level of t2 e-cigarette use was equivalent to change in e-cigarette use from t0 to t2, making the latent t2 e-cigarette intercept a measure of change in e-cigarette frequency. The latent t2 cigarette intercept also represents change in cigarette frequency between t0 and t2 because it is adjusted for pre-baseline smoking. Most importantly, if t2 e-cigarette use predicts t2 cigarette use, it implies that change in e-cigarette frequency predicts change in cigarette frequency at t2 (i.e. product substitution). Finally, any further linear change, in e-cigarette frequency after t2 is represented by the e-cigarette slope and thus, if the e-cigarette slope predicts cigarette slope, this provides more evidence of subject-level substitution.

We also incorporated an interaction between t0 cigarette frequency and t2 e-cigarette frequency, depicted in Figure 1 as an arrow from t0 cigarette frequency to the association between change in e-cigarettes and cigarettes at t2. To help with interpretation, t0 cigarette frequency was mean-centered so that the estimated main effect of latent t2 e-cigarette intercept (uptake) on latent t2 cigarette intercept (change from t0 to t2) would be for the average smoker.

The dual process model was estimated using maximum likelihood in Mplus (version 8.6)^16^. All standard errors were based on a robust, sandwich style correction for departures from multi-normality^17^. As a sensitivity check for the key path coefficients due to the modest sample size, the dual process growth model was re-estimated using a fully Bayesian approach with non-informative priors. This approach can bolster confidence in estimated fixed effects such as path coefficients because the entire posterior distribution of a fixed effect is simulated and inspected to perform hypothesis testing^18^.

## RESULTS

### Descriptive analysis of cigarette and e-cigarette use

We plotted growth curves for cigarettes and e-cigarettes for each study participant in the e-cigarette arm (Supplementary file Figure). Participants were sorted by magnitude of change in cigarette use between baseline and 8 weeks, with decreasing amounts of change moving to the right and down the rows. The predominant change pattern shows decreases in cigarette use associated with increases in e-cigarette use (where the black and red lines cross) in the first 2 weeks of the trial (e.g. see row 1, panels 1–7). There were also some participants for whom increased e-cigarette use did not herald much change in cigarette smoking (row 8, panels 1, 4, and 7).

With respect to e-cigarette use, there were quick adopters whose e-cigarette use changed rapidly by t2 (row 1, panels 1 and 5), others whose e-cigarette use climbed throughout the trial (row 1, panels 9, 11, and 12), and a few who had little e-cigarette uptake (row 9, panels 9–11). Most maintained a low level of cigarette use, along with a high level of e-cigarette use during the trial, but there were some who reported no cigarette use after t2 (row 1, panels 1 and 12), and one who reported no use of either product after t4 (row 1, panel 6). Finally, the degree of change seemed to be correlated with the intensity of cigarette smoking at baseline; that is, those with higher t0 cigarette smoking intensity tended to also have higher intensity of t2 e-cigarette use and lower intensity of t2 cigarette use.

### Product substitution between adjacent time points

**Figure 2:**
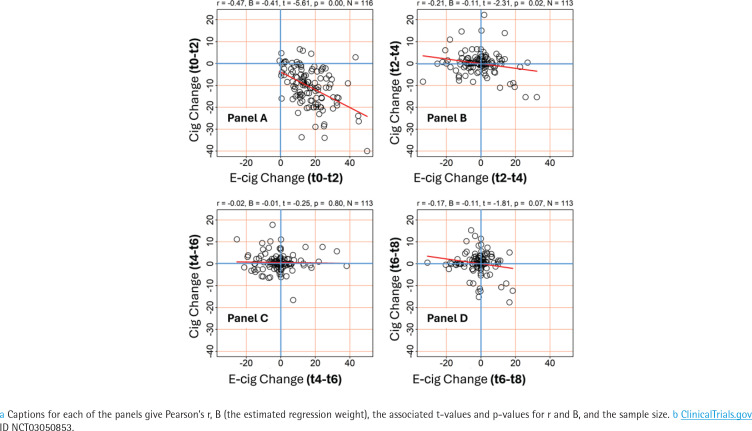
Scatterplots showing the association ^a^ between change in e-cigarette use and change in cigarette use between different adjacent time points throughout the trial, participants (N=120) in the treatment arm of the trial ^b^, recruitment period March 2017 through August 2020

Figure 2 shows scatterplots of the relationship between change in e-cigarette use (x-axis) and change in cigarette use (y-axis) between adjacent time points in the trial. The axes on the plots are shifted to include negative values for e-cigarettes and positive values for cigarettes, to accommodate individuals for whom e-cigarette use declined or cigarette use increased during later periods of the trial. Each subplot can be divided into four quadrants using the horizontal grid line at 0 and the vertical grid line at 0, both indicated by blue lines. The top right quadrant for example is to the right of the vertical grid line at 0 and above the horizontal grid line at 0; change in this quadrant is positive for both variables.

Figure 2 Panel A shows the predominant increase in e-cigarettes and decrease in cigarettes (negative correlation); most points are in the lower right quadrant, illustrating the negative correlation between change in e-cigarette sessions between t0 and t2 weeks and change in cigarettes per day (Pearson’s r= -0.47, β= -0.41) between those two time points. This indicates that the unadjusted substitution effect was about -0.4 cigarettes for each additional e-cigarette session. Panels B–D showed little change, with most points clustered near the origin (0,0). As shown by the fitted lines, the correlation between e-cigarette and cigarette use was much lower in Panels B–D (e.g, only 0.02 in Panel C) and the fitted lines were much less steep (e.g, β= -0.01 in Panel C), with most of the spread along the horizontal axis, as if some e-cigarette users were adjusting their intensity of use without much change in their cigarette consumption.

### Stratification by cigarette smoking intensity

**Figure 3:**
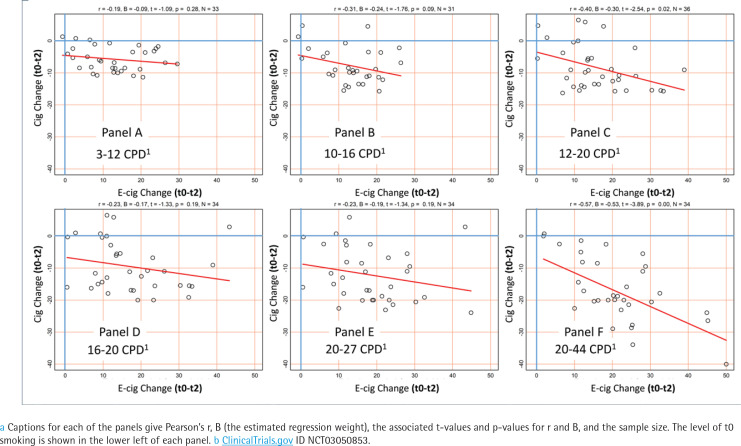
Scatterplots showing the association ^a^ between change in e-cigarette use and change in cigarette use between baseline (t0) and two weeks (t2), by t0 cigarette smoking intensity (partially overlapping strata), participants (N=120) in the treatment arm of the trial ^b^, recruitment period March 2017 through August 2020

Figure 3 shows the association between change in e-cigarette frequency and change in cigarette frequency between t0 and t2, stratified by t0 cigarette frequency (partially overlapping strata). The red lines show linear regression best fit plots, with relevant regression statistics in the top margin (B representing the average decrease in cigarettes per day for each additional e-cigarette session). Viewing the lines sequentially from Panel A through Panel F illustrates that, as t0 cigarette frequency increased, there was a tendency toward a stronger negative association, indicating greater product substitution. For lighter smokers, B was 0.09, -0.24, -0.30, -0.17, -0.19, and -0.53 for Panels A, B, C, D, E, and F, respectively. Although it is not clear from this analysis that the pattern of changes was statistically significant, the findings suggest an interaction between baseline cigarette smoking intensity and the level of product substitution. Additionally, it is notable that, even for light smokers (Panel A), t2 e-cigarette intensity ranged from little use to upwards of 20 e-cigarette sessions per day during the first two weeks of the trial.

### Fitting a dual process growth model

Figure 1 shows key pathways in the linear dual process growth model with estimates shown only for significant associations. Overall model fit statistics are not available for models that include a latent variable in an interaction. Model fit prior to adding the interaction term, however, was adequate [χ^2^=61.8, df=32, p=0.0012, RMSEA (root mean squared error of approximation)=0.088, CFI (Bentler’s comparative fit index)=0.942, TLI (Tucker-Lewis fit index)=0.935]. Full results for the model can be found in Supplementary file Table 2.

The frequency of e-cigarette sessions at t2 increased by 0.27 (95% CI: 0.02–0.51) sessions for each additional t0 cigarette. There was a significant interaction (p=0.0033) between t0 cigarette use and the strength of the association between t0 and t2 change in e-cigarette frequency and t0 and t2 change in cigarette use; the association became stronger in the negative direction by -0.03 for each additional t0 daily cigarette.

The main association of interest (arrow from t2 e-cigarettes to t2 cigarettes) represents the substitution effect for an average (18 cigarettes per day) t0 smoker; t2 cigarette frequency decreased by -0.39 (95% CI: -0.56 – -0.22) cigarettes for each additional e-cigarette session. Finally, there were no significant associations for the subsequent changes after 2 weeks in cigarette or e-cigarette frequency during the remaining 6 weeks when participants were offered e-cigarettes in the trial.

The implications of effect modification are detailed in Table 1, which shows how the estimate for level of t0-t2 product substitution changed with various t0 cigarette smoking frequencies. For those with t0 cigarette frequency of <10, the B was very small, between 0 and -0.09 (decrease of 0.09 cigarettes for each additional e-cigarette session) and not statistically significant, but for the heaviest smokers (38 cigarettes per day), the level approached one-for-one product substitution (p<0.0001).

**Table 1:** Effect modification: t0 cigarette frequency affects the level of product substitution between t0 and t2, participants (N=120) in the e-cigarette arm of the trial ^a^, recruitment period March 2017 through August 2020

*Cigarettes per day*	*3*	*8*	*13*	*18*	*23*	*28*	*33*	*38*
Distribution percentile	0.01	0.07	0.3	0.53	0.81	0.88	0.92	0.96
β^b^	0.01	-0.12	-0.26	-0.39	-0.52	-0.65	-0.79	-0.92
95% CI	-0.25–0.26	-0.32–0.07	-0.42 – -0.1	-0.56 – -0.21	-0.74 – -0.29	-0.94 – -0.36	-1.14 – -0.41	-1.36 – -0.92

a ClinicalTrials.gov ID NCT03050853.

b Beta for the association between change in e-cigarette use and change in cigarette use between Week 0 and Week 2, row below shows the 95% confidence interval for this estimate.

### Sensitivity analysis

The Bayesian simulation procedure does not depend on large sample assumptions and works in small samples. For the structural paths, the pattern of statistical significance across 4 standard categories (0 < p ≤ 0.001; 0.001 < p ≤ 0.01; 0.01 < p ≤0.05; and 0.05 < p ≤1) was identical and the estimates were very similar to results obtained by robust maximum likelihood.

## DISCUSSION

We described several salient characteristics of tobacco use behavior during this e-cigarette substitution trial. Increases in e-cigarette use were moderately associated with decreases in cigarette use, and almost all product substitution occurred during the first 2 weeks of the trial, despite the ongoing provision of e-cigarettes for the full 8 weeks. These findings are consistent with another substitution trial in which almost all substitution occurred in the first two weeks^5^.

A 2016 NIH workshop on e-cigarettes concluded that prospective clinical trials are needed to ‘understand whether e-cigarettes have value in harm reduction by leading to a complete switch from conventional cigarettes to e-cigarettes.’^19^. The present findings, if replicated, suggest that short-term (2-week) comparative effectiveness trials could be developed in which cigarette smokers are randomized to multiple different potentially lower harm products. The metric proposed in this study, the level of early product substitution, has face validity as a clinically relevant objective outcome measure. If product-level differences in early product substitution are found, this information could guide users toward products that maximize substitution. This outcome would be even more clinically relevant if early product substitution was linked with harm reduction outcomes like exhaled CO or urinary carcinogens later in the trial.

Short-term trials have other advantages. Adaptive treatment (SMART) trials^20^ could be developed in which cigarette smokers who exhibit little product substitution during the first 2-week period are then randomized to different lower harm product. These types of designs would be a much more efficient use of resources than the current approach, which typically involves a 4-week to 12-week e-cigarette provision period, only one or two treatment products, and often with an assessment-only control group.

Another goal of these trials could be to identify subgroups for whom substitution is more effective. In the present trial, heavier cigarette smoking frequency at t0 was associated with higher t2 e-cigarette and a stronger association between increases in e-cigarette use and decreases in cigarette use. The interaction effect is clinically interesting because it suggests that heavier smokers may be more willing and able to substitute e-cigarettes for cigarettes. Given strong evidence that heavier smoking is associated with poor cessation outcomes^21^, this finding has great relevance to the potential for e-cigarette substitution as a harm reduction approach especially for heavy smokers who cannot quit with standard evidence-based cessation treatment. In this trial, e-cigarette substitution was not effective in promoting product substitution among light smokers; if confirmed, this may point to promoting standard cessation treatment in this group.

### Limitations

This study used only self-reports of cigarette and e-cigarette use, which are imperfect measures of consumption^22^ and could bias the findings. Growth models could be designed to incorporate other measures of use, like CO, smoking topography, returns of e-cigarette cartridges and used cigarette filters to increase the reliability of the latent constructs that model uses. Although the measurement of short-term correlated change seems like a clinically meaningful measure of e-cigarette appeal, it is not clear how this metric relates to longer term harm reduction, and this could also be investigated to further validate the measure. We know of no other studies of this nature and recognize that the model presented here could be sample-specific and should be subjected to confirmation in independent samples from other substitution trials. Finally, the substitution effect in this trial applies not to a single product but to an e-cigarette brand that offered two flavors and two nicotine concentrations as well as smoker’s own brand of combustible cigarettes.

## CONCLUSIONS

This study found that smokers implemented most of their substitution behavior, in which increase in e-cigarette consumption was correlated with reduction in cigarette consumption, within the first two weeks of the trial. The study also found that heavier users of cigarettes not only reported more e-cigarette sessions, but also greater substitution, approaching one-for-one product replacement. If the dynamics of product substitution in this trial are replicated, trials that compare multiple lower harm products may be feasible.

## Supplementary Material



## Data Availability

The data supporting this research are available from the authors on reasonable request.
