# Supplementary material for: Level and timing of product substitution in a trial of e-cigarettes for smokers not interested in quitting
Source: Tob Induc Dis. 2024 Jun 13;22:10.18332/tid/189220. doi: 10.18332/tid/189220 (PMC11170977; doi:10.18332/tid/189220)
Supplement: Supplementary file 1 [file TID-22-104-s1.pdf]

How the exploratory data analysis informed and modified our thinking about modeling substitution.

The use of dual process models to examine product switching was proposed a priori in a 2016 grant proposal (available on request from JDS). Thus, most of the design elements of the dual process growth model were based on considerations of the intervention design. However, some considerations were made based on the preliminary analysis described in Figures 2 and 3. Those design elements are described below because they involve a melding of methods and results sections.

After initial exploratory analyses (scatter plots and bivariate correlations described in the first part of the results section) revealed that most product substitution occurred between baseline (t0) and the week 2 (t2) follow up, we considered but rejected dual quadratic growth models as being too complex to be supported by the modest sample size. We also considered but rejected an exclusive focus on t0 to t2 (or any other single follow up point) as being incomplete. Because baseline e-cigarette use was essentially zero in the current sample, we settled on using baseline cigarette frequency as an exogenous covariate in a dual linear growth model from t2 to the final follow up at 8 weeks (t8) with structural relations between latent intercepts and slopes, adjusted for t0 smoking as illustrated in Figure 1.

We started a priori with the standard statistical assumptions including that all predictive relations would be linear and additive. Upon checking, however, initial analyses suggested that t0 cigarette frequency (cig/d) moderated the effect of t2 e-cigarette session frequency (e-cig/d) on t2 cig/d, with higher t0 smoking frequency being associated with more substitution. We incorporated it as an interaction between an observed variable (t0 cig/d) and the effect of a latent variable (t2 e-cig/d), depicted in Figure 1 as a horizontal arrow from t0 cig/d to the association between t2 e-cig/d and t2 cig/d. We also considered but rejected other options due to the added complexity, for example a log transformation of cigarette frequency either directly to the raw data or incorporated in the model estimation framework (e.g., a poisson regression with a log link).

Supplementary Table 1. Baseline demographics of the treatment and assessment only groups, participants (N=240) in the Switchit trial,[1] recruitment period 03-2017 through 08-2020.

Characteristics	No. (%) of participants			χ^2	*p*-value
	Total (*N* = 240)	E-cigarette (*n* = 120)	Assessment (*n* = 120)		
High school graduate or GED	174 (72.5)	91 (52.3)	83 (47.7)	1.34	.25
Male gender	125 (52.1)	60 (50.0)	65 (54.2)	0.42	.52
Race[a]				3.63	.60
White	130 (54.2)	65 (54.2)	65 (54.2)		
Black	79 (32.9)	40 (33.3)	39 (32.5)		
Mixed and other	30 (12.5)	15 (11.7)	16 (13.3)		
Hispanic ethnicity	24 (10.0)	12 (10.0)	12 (10.0)	0.00	1.00
Ever married	95 (39.6)	51 (42.5)	44 (36.7)	0.85	.36
Number of cigarettes				0.02	.69
Less than 19 per day	139 (57.9)	71 (59.2)	68 (56.7)		
20 or more per day	101 (42.1)	52 (43.3)	49 (40.8)		
Diagnosis				0.02	.90
Schizophrenia spectrum	113 (47.1)	57 (47.5)	56 (46.7)		
Bipolar disorder	127 (52.9)	63 (52.5)	64 (53.3)		
	Mean ± SD	Mean ± SD	Mean ± SD	*T* test	*p*-value
Age	45.9 ± 11.9	46.3 ± 11.4	45.5 ± 12.5	0.60	.60
Carbon monoxide (ppm)	26.9 ± 19.9	25.8 ± 17.8	27.9 ± 21.9	0.83	.41
FTCD score	6.8 ± 1.5	6.7 ± 1.6	7.0 ± 1.5	1.30	.20
Brief Psychiatric Rating Scale score	47.8 ± 14.2	47.1 ± 14.5	48.6 ± 13.8	0.80	.60
Age at first hospitalization	21.6 ± 19.7	20.9 ± 10.0	22.3 ± 9.3	1.14	.26

FTCD, Fagerström Test for Cigarette Dependence; GED, General Education Development.
[a]One participant in the e-cigarette group refused to report racial category.

[1] Taken from: Pratt SI, Ferron JC, Brunette MF, Santos M, Sargent J, Xie H. E-Cigarette Provision to Promote Switching in Cigarette Smokers With Serious Mental Illness-A Randomized Trial. Nicotine Tob Res. Aug 6 2022;24(9):1405-1412. doi:10.1093/ntr/ntac082; ClinicalTrials.gov ID NCT03050853.

Supplemental Table 2: Full Figure 1 model results,[1] participants (N=120) in the treatment arm of the Switchit trial,[2] recruitment period 03-2017 through 08-2020.

effect	Descriptive labels for parameters	variable	estimate	std. error	z-value	p-value	95% CI low	95% CI high
Mean	cig intercept	CIGI	14.578	1.71	8.54	0.0000	11.23	17.92
Mean	cig slope	CIGS	0.244	0.43	0.56	0.5733	-0.60	1.09
Mean	ecig intercept	ECIGI	16.831	0.89	18.83	0.0000	15.08	18.58
Mean	ecig slope	ECIGS	-0.754	1.10	-0.68	0.4944	-2.92	1.41
Mean	t0 cigs	CIG0	0.000	0.78	0.00	0.9999	-1.52	1.52
Regression	cig intercept on ecig intercept	CIGI on ECIGI	-0.391	0.09	-4.53	0.0000	-0.56	-0.22
Regression	cig intercept on ecig intercept by cig0 interaction	CIGI on ECGICG0	-0.026	0.01	-2.94	0.0033	-0.04	-0.01
Regression	cig intercept on cig0	CIGI on CIG0	1.027	0.19	5.55	0.0000	0.66	1.39
Regression	cig slope on cig intercept	CIGS on CIGI	-0.011	0.02	-0.51	0.6087	-0.06	0.03
Regression	cig slope on ecig intercept	CIGS on ECIGI	-0.005	0.02	-0.26	0.7968	-0.04	0.03
Regression	cig slope on ecig slope	CIGS on ECIGS	-0.127	0.09	-1.41	0.1596	-0.30	0.05
Regression	cig slope on cig0	CIGS on CIG0	-0.018	0.02	-1.10	0.2713	-0.05	0.01
Regression	ecig intercept on cig0	ECIGI on CIG0	0.267	0.13	2.12	0.0341	0.02	0.51
Regression	ecig slope on cig intercept	ECIGS on CIGI	0.047	0.05	0.93	0.3545	-0.05	0.15
Regression	ecig slope on ecig intercept	ECIGS on ECIGI	-0.007	0.05	-0.14	0.8919	-0.10	0.09
Regression	ecig slope on cig0	ECIGS on CIG0	-0.057	0.04	-1.43	0.1521	-0.14	0.02
Variance	cig intercept	CIGI	26.401	5.61	4.71	0.0000	15.40	37.40
Variance	cig slope	CIGS	0.197	0.15	1.34	0.1812	-0.09	0.49
Variance	ecig intercept	ECIGI	64.370	13.94	4.62	0.0000	37.06	91.68
Variance	ecig slope	ECIGS	2.019	0.55	3.64	0.0003	0.93	3.11
Variance	cig time specific residual	CIG2	11.613	2.10	5.52	0.0000	7.49	15.73
Variance	ecig time specific residual	ECIGT2	41.241	5.86	7.04	0.0000	29.75	52.73
Variance	t0 cigs	CIG0	72.123	10.58	6.82	0.0000	51.38	92.87

[1] p-level color-code scheme: white = .05 < p < 1, pink = .01 < p <= .05, orange = .001 < p <= .01, green = 0 < p <= .001.

[2] ClinicalTrials.gov ID NCT03050853

Figure Legend

Supplementary Figure. Dual growth curve plots, observed data for cigarettes per day (black lines) and e-cigarette sessions per day (red lines) by week for each of the 120 participants in the treatment arm of the Switchit trial,[1] recruitment period 03-2017 through 08-2020.

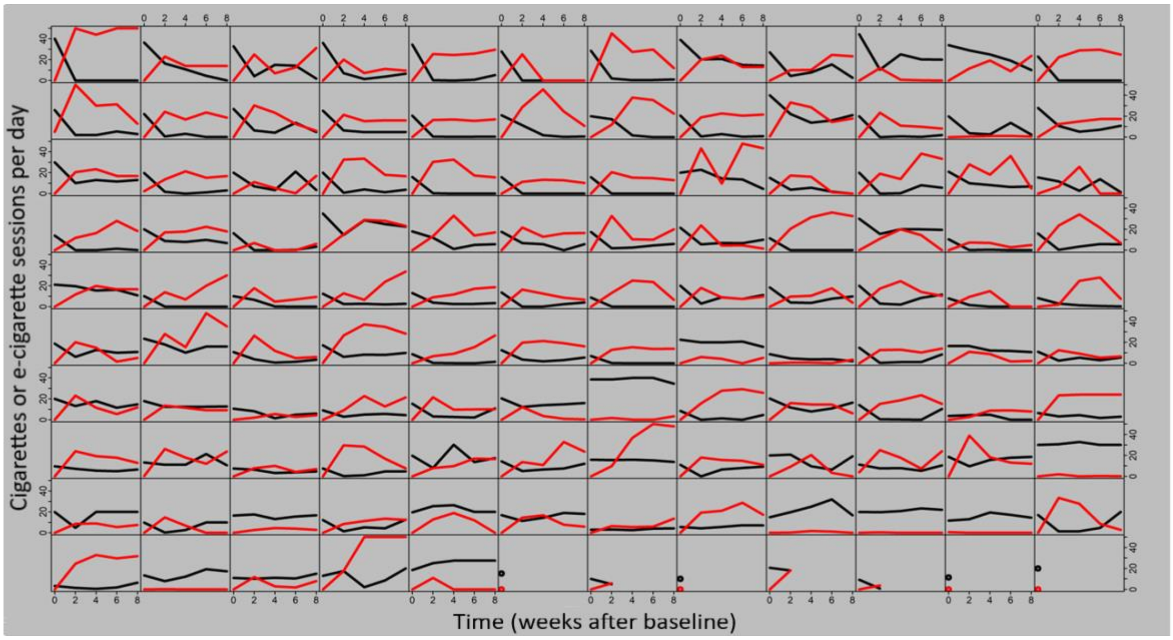

[1] ClinicalTrials.gov ID NCT03050853.

© 2024 Sargent J.D. et al.
